# Genetic dissection of flag leaf morphology in wheat (*Triticum aestivum* L.) under diverse water regimes

**DOI:** 10.1186/s12863-016-0399-9

**Published:** 2016-06-28

**Authors:** Delong Yang, Yuan Liu, Hongbo Cheng, Lei Chang, Jingjing Chen, Shouxi Chai, Mengfei Li

**Affiliations:** Gansu Provincial Key Lab of Aridland Crop Science/School of Life Science and Technology, Gansu Agricultural University, Lanzhou, 730070 China; School of Agronomy, Gansu Agricultural University, Lanzhou, 730070 China

**Keywords:** *Triticum aestivum*, Drought stress, Flag leaf morphology, Quantitative trait Loci, Genetic dissection

## Abstract

**Background:**

Morphological traits related to flag leaves are determinant traits influencing plant architecture and yield potential in wheat (*Triticum aestivum* L.). However, little is known regarding their genetic controls under drought stress. One hundred and twenty F_8_-derived recombinant inbred lines from a cross between two common wheat cultivars Longjian 19 and Q9086 were developed to identify quantitative trait loci (QTLs) and to dissect the genetic bases underlying flag leaf width, length, area, length to width ratio and basal angle under drought stress and well-watered conditions consistent over four environments.

**Results:**

A total of 55 additive and 51 pairs of epistatic QTLs were identified on all 21 chromosomes except 6D, among which additive loci were highly concentrated in a few of same or adjacent marker intervals in individual chromosomes. Two specific marker intervals of Xwmc694-Xwmc156 on chromosome 1B and Xbarc1072-Xwmc272 on chromosome 2B were co-located by additive QTLs for four tested traits. Twenty additive loci were repeatedly detected in more than two environments, suggestive of stable A-QTLs. A majority of QTLs involved significant additive and epistatic effects, as well as QTL × environment interactions (QEIs). Of these, 72.7 % of additive QEIs and 80 % of epistatic QEIs were related to drought stress with significant genetic effects decreasing phenotypic values. By contrast, additive and QEIs effects contributed more phenotypic variation than epistatic effects.

**Conclusions:**

Flag leaf morphology in wheat was predominantly controlled by additive and QEIs effects, where more QEIs effects occurred in drought stress and depressed phenotypic performances. Several QTL clusters indicated tight linkage or pleiotropy in the inheritance of these traits. Twenty stable QTLs for flag leaf morphology are potentially useful for the genetic improvement of drought tolerance in wheat through QTL pyramiding.

## Background

Wheat (*Triticum aestivum* L.), one of the most important foodstuff crops in the world, is grown under a broad range of environmental conditions in terms of water regimes, climatic factors, and soil types. As current changes in global climate have increased variability in precipitation with more frequent episodes of drought [1], wheat production in semiarid and arid regions is increasingly constrained due to erratic drought stresses [2]. In particular, terminal drought occurring during the reproductive phase is responsible for poor grain set and development and finally results in substantial reductions in grain yield [3]. Therefore, the improvement in drought tolerance, as well as grain yield, is of very importance in the selection of wheat cultivars in dryland environments.

Grain yield in cereal crops is due to complex physiological and biochemical processes but is essentially associated with the carbohydrate accumulation process of grain filling, which in turn is attributed to leaf functionalities [4]. By contrast to other leaves in the duration of reproductive phase, flag leaves are the main organ for photosynthesis, providing the major assimilate source required for plant growth and panicle development and also sensing environmental signals for adaptation [4, 5]. For example, under favorable conditions and depending on wheat genotype, the wheat flag leaf contributes 45–58 % of photosynthetic performance [6] and 41-43 % of assimilates used in grain filling after flowering [7]. In this regard, key components underlying grain yield in cereal crops are positively correlated with flag leaf size estimated by length (FLL), width (FLW) and area (FLA) [8–12], flag leaf length to width ratio (FLWR) [13] and basal angle of flag leaf (BAFL) [14, 15]. Based on this, improvement of flag leaf traits has led to a large increase in grain yield [16]. Under drought conditions, water deficit in cereal crops significantly decreases leaf areas and adjusts the BAFL to avoid excessive transpiration loss [17]. Positive adaptation may also delay leaf senescence induced by drought stress, thus maintaining photosynthetic capacity and a favorable supply of assimilates to the grain for a longer period of time to assure better grain yield [18]. As a result, wheat genotypes with smaller and more erect flag leaves are considered more able to roll their leaves to reduce water loss in response to drought stress than genotypes with lax leaves [19], resulting in higher yields [20]. Qian et al. [21] also found that, in wheat plants exposed to drought stress, FLW, FLL and BAFL during grain-filling were positively correlated with yield component traits, but the correlation coefficients were smaller than those under well-watered conditions [21]. Of course, it is indisputable that reduction in flag leaf area induced by drought stress is *per se* conductive to limited water use and could also result in lower productivity [22], whereas ideal flag leaf sizes and shapes in wheat are still beneficial for sustaining yield potential in water-deficit environments [15, 19]. Therefore, obtaining optimal flag leaf morphology (FLM) could be an important target in breeding wheat for drought tolerance, especially under terminal drought stresses.

To better develop molecular marker-assisted selection and explore novel functional genes for FLM in improving drought tolerance in wheat, it is essential to dissect the molecular genetic basis. This understanding will provide knowledge on how genes/QTLs underlying phenotypic variation are modulated. Much effort has already been exerted to uncover the genetic mechanism for such traits in cereal crops [5, 23–25]. Early studies showed that FLM-related traits were under additive control combined with partial dominance and epistasis [14, 26], or even predominantly controlled by complex epistatic interactions, dominance, and additive × dominance variation [7]. Furthermore, the phenotypic variation was governed by one gene with at least three distinct alleles [27]. With the recent availability of molecular markers and genetic maps, a large number of quantitative trait loci (QTLs) for FLM-related traits have been identified in wheat [15, 24, 28–32], rice [10–12, 33–35] and barley [23, 25]. Two major QTLs (*qFLL1* and *qFLW4*) for FLL and FLW in rice were fine mapped [11, 35], and even some genes controlling FLW are cloned [36]. In wheat, putative QTLs with flexible expressions in various genetic populations and environments have been detected on almost all 21 chromosomes. For example, using a wheat recombinant inbred line (RIL) population, Fan et al. [32] identified 38 additive QTLs (A-QTLs) for FLW, FLL and FLA on 12 chromosomes, explaining 3.96–27.68 % of the phenotypic variance. However, only three A-QTLs were stable across environments [32]. Working on another RIL population, Wu et al. [24] found that just four of 61 A-QTLs were repeatedly expressed in all environments [24]. Isidro et al. [15] detected 30 A-QTLs for BAFL on chromosomes 2A, 2B, 3A, 3B, 4B, 5B and 7A in a double haploid (DH) population, individually accounting for 8.9–37.2 % of the phenotypic variance. That study confirmed that the pattern of QTL expression was dynamic and time-dependent during the ontogeny of BAFL [15]. Recently, one of the major QTL for FLW, *QFlw.nau-5A.1*, was fine mapped to a 0.2 cM Xwmc492-Xwmc752 interval in the chromosome 5AL 12-0.35-0.57 deletion bin [30], closely linked with *Fhb5*, a gene for type I Fusarium head blight resistance [29, 30, 37]. Some important chromosome regions with abundant QTL information for FLM-related traits overlapped the marker intervals of QTLs associated with yield component traits [8, 28, 29, 31, 32]. These findings further confirmed that FLM is quantitatively inherited by ploygenes and is significantly influenced by environmental factors. However, few studies so far have been undertaken to fully dissect the variability in genetic components and QTL × environment interactions (QEIs) under the drought stress.

In this study, a RIL population of 120 F_8_-derived lines grown under drought stressed (DS) and well-watered (WW) regimes in four environments was employed to map QTLs for FLM-related traits FLL, FLW, FLWR, FLA and BAFL. The objectives were to identify A-QTLs and epistatic QTLs (AA-QTLs) underlying components of FLM-related traits and to analyze additive QEIs (A-QEIs) and epistatic QEIs (E-QEIs) of the traits in two water regimes across environments. The findings might provide a better understanding of the genetic mechanisms governing FLM-related traits in wheat under water-limited environments, and should benefit genetic improvement of drought tolerance in wheat by pyramiding favorable QTLs.

## Methods

### Plant materials

A RIL population of 120 F_8_-derived lines was developed from a cross between two Chinese winter wheat varieties, Longjian 19 and Q9086. Longjian 19, released by the Gansu Academy of Agricultural Sciences, Lanzhou, Gansu, is an elite drought-tolerant cultivar widely grown in rainfed areas (300-500 mm annual rainfall) in northwestern China. Q9086, released by Northwest Agriculture & Forestry University, Yangling, Shanxi, is a high-yielding cultivar suitable for cultivation under conditions of sufficient water and high fertility, but is prone to early senescence under terminal drought stress. The two parents differ significantly in several agronomical and physiological traits under terminal drought stress, such as plant height, grain weight and accumulation and remobilization of water soluble carbohydrates in stems [38–40].

### Field trials

The RIL population and parents were grown at three locations in Gansu province, namely, at Yongdeng (103°18’ E, 36°42’ N, 2140 m above sea level) in 2011 (294.3 mm of annual rainfall, 1879.8 mm of annual evaporation capacity, 6.2 °C of average daily temperature) and 2012 (309.6 mm of annual rainfall, 1906.2 mm of annual evaporation capacity, 6.4 °C of average daily temperature); at Anning (103°51’ E, 36°04’ N, 1520 m above sea level) in 2012 (346.5 mm of annual rainfall, 1664.9 mm of annual evaporation capacity, 8.1 °C of average daily temperature), and at Yuzhong (104°07’ E, 35°51’ N, 1900 m above sea level) in 2013 (328.4 mm of annual rainfall, 1495.8 mm of annual evaporation capacity, 7.2 °C of average daily temperature). The environments were named E1, E2, E3 and E4, respectively. The experimental field in each year was divided into DS and WW sections. The DS treatment was equivalent to rainfed conditions with rainfall of 95.8, 98.6, 113.2 and 101.5 mm in E1 to E4, respectively, during the growing season (from early October in the sowing year to late June in harvesting year). The WW treatment involved irrigation with 750 m^3^ ha^-1^ water supply at each of pre-overwintering, jointing, and flowering stages, respectively. The field designs were randomized complete blocks with three replications. Each plot was 2 m long with six rows spaced 20 cm apart with approximately 160 plants per row. Nutrition supplied to all treatments was 180 kg ha^-1^ N, 20 kg ha^-1^ P_2_O_5_ and 75 kg ha^-1^ K_2_O only at sowing. Other aspects of field management followed the local practices.

Five FLM traits, FLL, FLW, FLWR, FLA and BAFL, were evaluated in this study. For each plot, the main shoots from 10 plants in the centre of each row were randomly selected to measure FLL, FLW and BAFL at the milky ripe stage (Feeks 11.1) and to investigate the plant height (PH), spikelet number (SN), kernel number (KN), kernel weight per spike (KW) of main shoots and yield per plant (YP) at the kernel ripe stage (Feeks 11.4). The FLL and FLW measurements were made at the longest and widest parts of the flag leaf using a ruler. The BAFL from the peduncle to the midrib of the flag leaf surface was determined with a protractor. FLA and FLWR were calculated as follows: FLA = FLL × FLW × 0.75 and FLWR = FLL/FLW. Agronomic traits were determined by conventional methods. Trait means of 10 samples from each plot were used in the data analysis based on three replications.

### Data analysis

Basic statistics and Pearson’s correlation analysis were performed on the phenotypic data from each water environment. Analysis of variance (ANOVA) was employed to evaluate the total and residual variances among RIL progenies for each FLM-related trait. Broadsense heritability (*h*^2^_B_) was estimated for each trait using ANOVA analysis and method proposed by Toker [41]. All analyses were performed using the SPSS version 18.0 statistical package and *P* values less than 0.05 were significant.

A genetic linkage map of 21 chromosomes, consisting of 524 simple sequence repeats (SSR) marker loci, was previously made for the RIL population [38, 39]. The map spanned 2266.7 cM with an average distance of 4.3 cM between adjacent markers and average 24.9 SSR markers per each chromosome. To dissect the quantitative genetic basis of FLM-related traits in the RIL population, the phenotypic data for the trait under both water regimes (DS and WW) as a set of variants in each environment were subjected to QTL analysis using the software QTLMapper version 1.0 set for composite interval mapping of a mixed linear model [42]. The genetic model divided genetic effects into additive effects (*A*), epistatic effects (*AA*), and QEIs (*AE* and *AAE*) effects. QTLs with genetic effects indicated that genes in these genomic regions were expressed in the same way across environments. QTLs with *AE* and *AAE* effects suggested that gene expression at those loci was environmentally dependent [42]. The closest marker to each local log odds (LOD) peak (putative QTL) was used as a cofactor to control the genetic background while testing at a position of the genome. The threshold LOD score to declare the presence of a QTL was 2.50, and the significance level was *P* < 0.005 for identifying additive and epistatic effects of QTLs and QEIs effects. If a QTL for one trait was detected repeatedly in two or more environments, it was considered a stable QTL. The QTL nomenclature was according to the rule “QTL+ trait + lab designation + chromosome”.

## Results

### Phenotypic variations

The phenotypic means for five FLM-related traits from the RIL population and parents, along with basic statistics under DS and WW conditions in four environments, are summarized in Table 1. Except for FLWR, the parents Longjian 19 and Q9086 differed significantly in the measured traits. Phenotypic means of Q9086 for FLL, FLW, FLA and BAFL were much higher than those of Longjian 19. Across all treatments, the means of the RIL population were intermediate between those of the two parents, showing wide phenotypic variability. The corresponding coefficients of variation (CV) ranged from 13.51 to 38.25 % in DS and from 8.28 to 24.44 % in the WW conditions. Some lines had more extreme values than the parents, showing substantial transgressive segregation. All skewness and kurtosis values were less than 1.0 in all treatments, indicative of continuous variation and a quantitative genetic basis.

**Table 1:** Phenotypic performance of traits related to flag leaf morphology of main shoots in the parents and RIL population grown under two water regimes in different environments

Trait	Environment	Parent		RIL population					
		Longjian 19	Q9086	Mean	Minimum	Maximum	CV (%)	Skewness	Kurtosis
FLL	E1	11.29/14.05	14.84^**^/17.62^**^	13.47/15.29	8.28/11.25	18.74/20.36	38.25/22.15	0.04/0.28	–0.07/0.14
	E2	13.61/17.43	17.29^**^/22.43^**^	15.68/22.57	10.70/17.49	21.54/28.08	32.57/21.74	0.32/0.30	0.62/–0.01
	E3	18.48/19.73	22.15^**^/24.76^**^	20.45/22.50	15.52/19.36	25.92/25.67	25.05/14.19	0.67/0.21	0.42/0.86
	E4	14.73/16.45	16.61*/20.72^**^	16.09/18.31	11.54/14.73	21.33/22.17	29.41/18.84	–0.06/–0.44	0.05/–0.04
FLW	E1	1.06/1.28	1.36^**^/1.49^**^	1.24/1.38	1.03/1.21	1.50/1.63	13.51/10.75	0.02/0.35	0.07/0.19
	E2	1.17/1.32	1.45^**^/1.51^**^	1.33/1.58	1.13/1.47	1.53/1.81	14.16/9.62	0.63/0.33	0.56/–0.38
	E3	1.35/1.56	1.55*/1.73*	1.44/1.59	1.20/1.43	1.73/1.79	15.46/8.28	0.21/0.33	–0.28/–0.34
	E4	1.23/1.34	1.36^**^/1.62^**^	1.32/1.45	1.15/1.33	1.52/1.58	14.20/8.57	0.08/0.34	0.03/0.04
FLWR	E1	10.65/10.98	10.91/11.83	10.90/11.15	8.15/9.08	13.71/13.23	25.38/17.87	–0.12/0.04	0.62/0.46
	E2	11.63/13.2	11.92/14.85	11.86/14.4	8.91/12.16	14.94/16.72	27.11/15.63	–0.47/0.60	0.59/0.92
	E3	13.69/12.65	14.29/14.31	14.19/14.33	11.18/12.34	17.06/16.21	19.28/12.78	–0.26/0.01	0.10/0.33
	E4	11.98/12.28	12.21/12.79	12.16/12.65	9.64/10.59	14.75/15.03	20.91/16.45	–0.62/–0.86	0.43/0.81
FLA	E1	8.98/13.49	15.14^**^/19.69^**^	12.64/15.92	8.08/13.20	16.31/21.65	29.86/18.31	0.42/0.69	–0.48/0.37
	E2	11.94/17.26	18.80^**^/25.40^**^	15.88/24.05	9.86/16.18	20.32/31.14	28.61/17.52	0.48/0.24	0.58/–0.36
	E3	18.71/23.08	25.75^**^/32.13^**^	22.27/27.09	16.64/20.24	27.81/35.61	19.74/15.11	0.41/0.64	–0.11/0.63
	E4	13.59/16.53	16.94*/25.17^**^	16.21/20.10	10.30/12.53	21.75/27.20	28.65/16.48	0.18/0.07	0.01/–0.36
BAFL	E1	31.56/43.74	43.45^**^/52.08*	35.70/48.80	23.24/34.86	49.37/61.79	35.12/24.44	0.38/0.47	0.07/–0.12
	E2	32.61/42.85	45.14^**^/53.37^**^	38.33/46.69	24.53/35.26	52.46/59.93	33.80/23.00	0.66/0.33	0.52/–0.04
	E3	37.64/49.02	49.53^**^/56.84*	41.24/50.17	29.18/37.91	52.24/60.36	25.28/19.25	0.56/0.39	0.58/0.87
	E4	33.64/45.43	46.39^**^/53.58*	37.32/49.34	25.74/37.23	50.18/61.56	28.68/22.70	0.20/0.09	–0.13/0.01

*FLL* flag leaf length, *FLW* flag leaf width, *FLWR* flag leaf length to width ratio, *FLA* flag leaf area, *BAFL* basal angle of flag leaf, *CV* coefficients of variation. E1 to E4 represent the location at Yongdeng (103°18’ E, 36°42’ N), Gansu, China, in 2011and 2012, at Anning (103°51’ E, 36°04’ N), Gansu, China, in 2012, and at Yuzhong (104°07’ E, 35°51’ N), Gansu, China, in 2013, respectively. Numbers at the left of the slash (“/”) are the phenotypic values of traits identified under DS, and numbers at the right for WW conditions; *and **mean significant difference in phenotypic values between the parents at 0.05 and 0.01 level, respectively

Results of ANOVA showed that the variances for phenotypic values in the RIL population reached the 0.05 or 0.01 significance levels, except for the interaction variances for both water regime × genotype and environment × water regime × genotype (Table 2). By contrast, variants of both water regime and environment had larger effects on the phenotypic variations of all traits, where their mean of squares ranged from 12.98 (FLW) to 80915.63 (BAFL) and were significantly higher than those for other variants. The phenotypic values of all five traits in the DS were significantly lower than those under the WW conditions (Table 1), indicating that DS might decrease the flag leaf size and narrow the BAFL relative to the peduncle. The estimated *h*^2^_B_ for all five traits varied from 0.48 to 0.62 (Table 2). Hence water environments made a significant impact on phenotypic variation and heritability of FLM-related traits.

**Table 2:** Analyses of variance (ANOVA) of traits related to flag leaf morphology of main shoots in the RIL population

Source of variation	*df*	FLL		FLW		FLWR		FLA		BAFL	
		MS	*F*	MS	*F*	MS	*F*	MS	*F*	MS	*F*
Environment (E)	3	6500.03	1502.11**	12.98	285.21**	1324.25	2712.94**	14330.06	833.21**	68449.95	17.60**
Water regime (W)	1	7570.59	1749.51**	20.74	1047.46**	442.60	906.74**	24166.66	1405.15**	80915.63	798.44**
Genotype (G)	119	19.43	6.34**	0.11	4.47**	10.42	27.50**	52.01	3.84**	495.72	6.59**
E × W	3	1068.64	246.96**	0.62	31.48**	259.05	530.70**	2389.20	138.92**	964.47	9.52**
E × G	357	14.61	3.38^*^	0.07	3.36^*^	6.48	13.28**	45.11	2.62*	350.67	3.46*
W × G	119	4.51	1.04	0.02	0.87	2.30	4.71	13.59	0.79	72.06	0.71
E × W × G	357	4.83	1.12	0.02	1.14	3.27	6.69	13.44	0.78	85.03	0.84
Error	1920	4.33		0.02		0.49		17.20		101.34	
*h* ^2^_B_		0.54		0.62		0.57		0.48		0.60	

*FLL* flag leaf length, *FLW* flag leaf width, *FLWR* flag leaf length to width ratio, *FLA* flag leaf area, *BAFL* basal angle of flag leaf, *MS* mean of square, *F*: *F* value estimated by ANOVA. *and **significant at 0.05 and 0.01 level, respectively

### Correlation analysis among tested traits

Correlations among all tested traits under both water regimes are given in Table 3. Most of the traits across environments were positively correlated with each other in two water regimes, with the exception of negative correlations between FLW and FLWR in both DS (*r* = –0.32^*^ to –0.54^**^) and WW conditions (*r* = -0.33^*^ to -0.46^**^). These positive correlations generally reached significant (*P* < 0.05) levels, with correlation coefficients (*r*) varying from 0.29^*^ to 0.93^**^. By contrast, correlation coefficients under DS (*r* = 0.31^*^to 0.93^**^) were commonly higher those under the WW conditions (*r* = 0.29^*^to 0.81^**^). Under both water regimes, FLL showed highly significantly positive correlations with FLA (*r* = 0.79^**^ to 0.93^**^/DS and 0.50^**^ to 0.81^**^/WW), FLWR (*r* = 0.61^**^to 0.75^**^/DS and 0.47^**^ to 0.70^**^/WW), and BAFL (*r* = 0.54^**^ to 0.79^**^/DS and 41^**^ to 0.62^**^/WW), respectively. FLA showed a similar degree of correlations to BAFL (*r* = 0.63^**^ to 0.82^**^/DS and 0.52^**^ to 0.71^**^/WW). This suggested that FLL was the main contributor to FLA and also influenced BAFL to some extent.

**Table 3:** Phenotypic correlation coefficients between traits related to flag leaf morphology of main shoots in the RIL population grown under two water regimes in different environments

Environment	Trait	FLL	FLW	FLWR	FLA	BAFL
E1	FLL		0.33*	0.70^**^	0.81^**^	0.62^**^
	FLW	0.41^**^		–0.38*	0.72^**^	0.46^**^
	FLWR	0.75^**^	–0.45^**^		0.35*	0.49^**^
	FLA	0.93^**^	0.81^**^	0.45^**^		0.71^**^
	BAFL	0.72^**^	0.64^**^	0.57^**^	0.82^**^	
E2	FLL		0.37^**^	0.52^**^	0.63^**^	0.60^**^
	FLW	0.43^**^		–0.46^**^	0.56^**^	0.45^**^
	FLWR	0.63^**^	–0.54^**^		0.35^**^	0.37^**^
	FLA	0.87^**^	0.81^**^	0.39^**^		0.65^**^
	BAFL	0.79^**^	0.54^**^	0.31*	0.71^**^	
E3	FLL		0.41^**^	0.48^**^	0.59^**^	0.41^**^
	FLW	0.46^**^		–0.33*	0.53^**^	0.40^**^
	FLWR	0.69^**^	–0.38^**^		0.42^**^	0.49^**^
	FLA	0.80^**^	0.65^**^	0.55^**^		0.56^**^
	BAFL	0.54^**^	0.47^**^	0.65^**^	0.63**	
E4	FLL		0.29*	0.47^**^	0.50^**^	0.53^**^
	FLW	0.34^**^		–0.43^**^	0.45^**^	0.39^**^
	FLWR	0.61^**^	–0.32*		0.32*	0.30*
	FLA	0.79**	0.78**	0.41**		0.52**
	BAFL	0.67**	0.44**	0.35**	0.64**	

*FLL* flag leaf length, *FLW* flag leaf width, *FLWR* flag leaf length to width ratio, *FLA* flag leaf area, *BAFL* basal angle of flag leaf. E1 to E4 represent the location at Yongdeng (103°18’ E, 36°42’ N), Gansu, China, in 2011and 2012, at Anning (103°51’ E, 36°04’ N), Gansu, China, in 2012, and at Yuzhong (104°07’ E, 35°51’ N), Gansu, China, in 2013, respectively. Correlation coefficients in the lower left segment apply to the DS, and those at the upper right part are for WW conditions; *and **significant at 0.05 and 0.01 level, respectively

Correlation coefficients between FLM-related traits and agronomic traits in the RIL population in different water environments were calculated (Table 4). Most correlations were weaker and non-significant. Correlation coefficients in DS conditions across environments were slightly lower than those in WW conditions. In both water regimes, SN was negatively correlated (*r* = –0.05 to –0.20^*^) with FLL, FLWR, FLA and BAFL, but was positively correlated (*r* = 0.04 to 0.13) with FLW. The other agronomic traits, such as PH, KN, KW and YP, showed positive correlations (*r* = 0.05 to 0.46^**^) with FLM-related traits, respectively. By contrast, PH showed higher positive correlations (*r* = 0.15 to 0.46^**^) with FLL, FLWR, FLA and BAFL. KW had stronger positive correlations with FLL (*r* = 0.26^*^ to 0.39^**^), FLWR (*r* = 0.19 to 0.33^**^) and FLA (*r* = 0.26^*^ to 0.40^**^). And, YP showed higher positive correlations with FLL (*r* = 0.18 to 0.32^**^) and FLA (*r* = 0.21^*^ to 0.38^**^), respectively.

**Table 4:** Phenotypic correlation coefficients between traits related to flag leaf morphology of main shoots and agronomic traits in the RIL population grown under two water regimes in different environments

Environment	Trait	PH	SN	KN	KW	YP
E1	FLL	0.34**/0.39**	–0.06/–0.14	0.18/0.19	0.27^*^/0.38**	0.22^*^/0.29**
	FLW	0.13/0.17	0.04/0. 08	0.08/0.10	0.06/0.08	0.09/0.10
	FLWR	0.30**/0.35**	–0.07/–0.11	0.15/0.17	0.24^*^/0.31**	0.12/0.14
	FLA	0.36**/0.41**	–0.08/–0.15	0.16/0.20^*^	0.26^*^/0.34**	0.21^*^/0.26^*^
	BAFL	0.16/0.20^*^	–0.05/–0.12	0.12/0.18	0.12/0.17	0.05/0.12
E2	FLL	0.35**/0.42**	–0.09/–0.17	0.16/0.22^*^	0.29^*^/0.33**	0.18/0.23^*^
	FLW	0.11/0.15	0.06/0.09	0.05/0.15	0.07/0.14	0.07/0.10
	FLWR	0.31**/0.36**	–0.08/–0.15	0.14/0.17	0.21^*^/0.29^*^	0.12/0.19
	FLA	0.37**/0.45**	–0.10/–0.18	0.12/0.19	0.28^*^/0.32^*^	0.23^*^/0.35**
	BAFL	0.15/0.22^*^	–0.07/–0.14	0.15/0.21^*^	0.13/0.21^*^	0.05/0.08
E3	FLL	0.39**/0.46**	–0.17/–0.20^*^	0.21^*^/0.30**	0.31**/0.39**	0.24^*^/0.32**
	FLW	0.16/0.20^*^	0.07/0.13	0.11/0.17	0.10/0.13	0.08/0.11
	FLWR	0.36**/0.41**	–0.11/–0.19	0.22^*^/0.25^*^	0.19/0.24^*^	0.15/0.22^*^
	FLA	0.38**/0.45**	–0.12/–0.14	0.19/0.28^*^	0.33**/0.40**	0.28^*^/0.38**
	BAFL	0.18/0.24^*^	–0.10/–0.16	0.17/0.22^*^	0.14/0.21^*^	0.10/0.23^*^
E4	FLL	0.30**/0.41**	–0.10/–0.17	0.14/0.21^*^	0.26^*^/0.35**	0.20^*^/0.24^*^
	FLW	0.12/0.16	0.05/0.09	0.09/0.14	0.08/0.10	0.08/0.12
	FLWR	0.32**/0.38**	–0.12/–0.13	0.12/0.23^*^	0.25^*^/0.33**	0.09/0.17
	FLA	0.34**/0.44**	–0.09/–0.16	0.15/0.24^*^	0.28^*^/0.35**	0.26*/0.27*
	BAFL	0.20*/0.25*	–0.07/–0.13	0.10/0.18	0.08/0.14	0.11/0.15

*FLL* flag leaf length, *FLW* flag leaf width, *FLWR* flag leaf length to width ratio, *FLA* flag leaf area, *BAFL* basal angle of flag leaf, *PH* plant height, *SN* spikelet number, *KN* kernel number, *KW* kernel weight per spike, *YP* yield per plant. E1 to E4 represent the location at Yongdeng (103°18’ E, 36°42’ N), Gansu, China, in 2011and 2012, at Anning (103°51’ E, 36°04’ N), Gansu, China, in 2012, and at Yuzhong (104°07’ E, 35°51’ N), Gansu, China, in 2013, respectively. Numbers at the left of the slash (“/”) are the correlation coefficients in DS and the numbers at the right refer to WW conditions; * and ** significant at 0.05 and 0.01 level, respectively

### Additive QTLs and water environmental interactions

A total of 55 A-QTLs governing FLM-related traits in environments E1 to E4 were mapped on chromosomes 1B, 2A, 2B, 3A, 4A, 4D, 5A, 5B, 6A, 6B and 7A, individually explaining 0.68 to 12.92 % of the phenotypic variation (Table 5). The number of QTL for each trait varied from 8 (BAFL) to 15 (FLW). Among them, 24 (43.6 %) loci involved favorable alleles from Q9086 increasing phenotypic values, whereas the other 31 (56.4 %) loci had favorable alleles from Longjian 19 for decreasing phenotypic values. This indicated that favorable alleles for FLM-related traits were almost equally dispersed between the parents. With regard to each trait, more favorable alleles (7-9 per trait) for FLW, FLWR and BAFL came from Longjian 19 and those for FLL and FLA were derived from Q9086. This implied that Q9086 contributed more genes regulating FLL and FLA in the RIL progenies, whereas Longjian 19 provided more genes controlling FLW and BAFL.

**Table 5:** Additive and interacting effects of QTL × environment of identified QTL for traits related to flag leaf morphology of main shoots in the RIL population

Trait	QTL	Flanking marker	Position (cM)	Environ.	*A*	*h* ^2^(*A*)%	*AE*	*h* ^2^(*AE*)%
FLL	*Qfll.acs–1B.1*	Xwmc694–Xwmc156	0	E1	0.48***	4.36	–0.41***	3.57
				E2	0.40***	3.84	–0.45***	4.24
				E3	0.43***	4.05	–0.43***	4.32
	*Qfll.acs–1B.2*	Xwmc367–Xgwm259	2	E1	0.36***	3.29	–0.40***	3.77
				E4	0.42***	4.35	–0.39***	3.26
	*Qfll.acs–2B.1*	Xbarc1072–Xwmc272	0	E1	0.50***	5.02	–0.51***	4.11
				E2	0.44***	4.18	–0.40***	3.63
	*Qfll.acs–3A.1*	Xwmc505–Xwmc343	0	E1	0.35***	4.12		
	*Qfll.acs–3A.2*	Xgwm162–Xmag3082	0	E4	0.41***	4.85	–0.42***	6.52
	*Qfll.acs–4A.1*	Xgwm165–Xmag1353	2	E3	–0.38***	3.26	0.37***	5.05
	*Qfll.acs–4D.1*	Xwmc489–Xgdm61	0	E2	–0.41***	5.64	–0.36***	6.00
				E3	–0.32***	4.45	–0.31***	4.93
				E4	–0.47***	4.64	–0.37***	6.59
	*Qfll.acs–5A.1*	Xgwm304–Xwm466	2	E2	0.54***	9.78	–0.39***	10.19
	*Qfll.acs–5A.2*	Xgwm205–Xgwm154	4	E2	–0.33***	3.68	0.33***	5.28
				E3	–0.40***	3.41	0.35***	4.12
	*Qfll.acs–5A.3*	Xgwm154–Xmag694	0	E4	–0.35***	3.62	–0.35***	3.86
	*Qfll.acs–5B*	Xbarc164–Xbarc4	2	E4	0.39***	3.75		
	*Qfll.acs–6B.1*	Xwmc182–Xmag2276	0	E2	–0.41***	5.58	0.32***	6.97
FLW	*Qflw.acs–2A.1*	Xwmc296–Xgwm122	0	E3	–0.02***	2.76	0.03***	9.83
				E4	–0.03***	2.91	0.03***	7.98
	*Qflw.acs–2A.2*	Xmag2150–Xgwm339	0	E3	–0.02***	2.12	0.02***	4.23
	*Qflw.acs–2B.1*	Xbarc1072–Xwmc272	0	E1	–0.04***	2.34	–0.04***	4.69
				E2	–0.02***	3.78	–0.02***	3.21
	*Qflw.acs–2B.2*	Xgwm630–Xksum248	4	E2	0.01***	1.03	0.01***	2.41
	*Qflw.acs–3A.1*	Xgwm162–Xmag3082	0	E3	0.03***	3.24	–0.02***	2.77
				E4	0.03***	7.90	–0.02**	4.25
	*Qflw.acs–3A.2*	Xwmc532–Xgwm674	2	E1	0.02***	0.75	–0.02***	1.81
	*Qflw.acs–3A.3*	Xwmc505–Xwmc343	0	E1	–0.02***	0.68	–0.02***	1.37
	*Qflw.acs–4D*	Xbarc92–Xwmc473	12	E2	–0.02***	2.06		
	*Qflw.acs–5A.1*	Xwmc492–Xgwm96	4	E1	–0.02***	2.51	–0.03***	3.04
				E2	–0.02***	3.45	–0.02***	4.63
				E3	–0.03***	4.04	–0.02***	2.77
				E4	–0.02***	3.15	–0.03***	3.82
	*Qflw.acs–5A.2*	Xcfa2185–Xbarc230	2	E2	0.03***	2.96	–0.03***	3.59
				E3	0.02***	2.87	–0.03***	2.61
	*Qflw.acs–5A.3*	Xbarc151–Xwmc630	0	E1	0.02***	3.05	–0.03***	3.05
				E3	0.03***	3.16	–0.02***	2.99
				E4	0.02***	3.08	–0.02***	3.21
	*Qflw.acs–5B.1*	Xbarc164–Xbarc4	0	E1	0.04***	3.31	–0.04***	5.75
	*Qflw.acs–5B.2*	Xwmc415–Xwmc508	10	E2	–0.02***	2.31	0.02***	4.13
	*Qflw.acs–6B*	Xwmc341–Xbarc198	0	E2	–0.02***	3.78	0.02***	5.71
	*Qflw.acs–7A.1*	Xbaec121–Xpsp3001	0	E4	–0.02***	3.15	–0.02***	3.94
FLWR	*Qflwr.acs–1B.1*	Xwmc694–Xwmc156	0	E1	0.35***	2.58		
	*Qflwr.acs–2A.1*	Xmag2150–Xgwm339	0	E3	0.37***	12.92		
	*Qflwr.acs–2A.2*	Xwmc522–Xwmc474	2	E3	–0.31***	9.41		
	*Qflwr.acs–2A.3*	Xgwm95–Xgwm249	2	E4	–0.23***	7.32		
	*Qflwr.acs–2B*	Xwmc272–Xgwm630	0	E1	–0.30***	2.20	0.22***	2.43
				E3	–0.24***	5.63		
	*Qflwr.acs–3A.1*	Xwmc505–Xwmc343	0	E1	–0.32***	2.64		
				E2	–0.32***	6.17	–0.34***	5.31
				E3	–0.20**	3.43		
				E4	–0.35***	2.87		
	*Qflwr.acs–3A.2*	Xwmc695–Xgwm162	4	E2	–0.35***	5.84		
	*Qflwr.acs–5A*	Xcfa2185–Xbarc230	2	E2	0.29***	3.13	–0.20***	3.47
				E3	0.33***	3.54		
				E4	0.25***	3.27	–0.29***	4.01
	*Qflwr.acs–6A.1*	Xwmc807–Xbarc1165	0	E4	–0.21**	6.29		
	*Qflwr.acs–7A.1*	Xpsp3001–Xgwm63	0	E4	–0.30***	4.14		
	*Qflwr.acs–7A.2*	Xwmc139–Xbarc195	2	E2	0.28***	3.41	–0.25***	4.83
FLA	*Qfla.acs–1B*	Xwmc694–Xwmc156	0	E1	0.55***	3.12	–0.60***	5.23
				E2	0.43***	2.58	–0.50***	4.09
				E4	0.40***	3.13	–0.38***	3.64
	*Qfla.acs–2B.1*	Xbarc1072–Xwmc272	0	E1	0.67***	3.70	–0.59***	3.39
				E3	0.61***	3.51	–0.51***	3.25
	*Qfla.acs–3A*	Xwmc695–Xgwm162	0	E1	–0.55***	3.01	–0.58***	3.96
				E4	–0.47***	1.83	–0.56***	5.23
	*Qfla.acs–4D.1*	Xbarc92–Xwmc473	6	E2	–0.51***	2.96	–0.31**	2.13
	*Qfla.acs–4D.2*	Xwmc489–Xgdm61	0	E4	0.52***	2.25	0.40***	2.75
	*Qfla.acs–5A*	Xwmc205–Xgwm154	4	E1	0.36***	4.48	–0.42***	3.84
				E3	0.40***	2.46	–0.45***	3.72
				E4	0.47***	3.29	–0.45***	3.60
	*Qfla.acs–5B*	Xwmc415–Xwmc508	10	E3	0.56***	1.83	–0.54***	3.36
	*Qfla.acs–6A.1*	Xwmc807–Xbarc1165	2	E4	0.63***	3.30	0.53***	4.79
	*Qfla.acs–6B*	Xbarc198–Xwmc182	4	E2	–0.48***	2.61	0.49***	5.34
BAFL	*Qbafl.acs–1B*	Xwmc694–Xwmc156	0	E4	1.52***	3.26	–1.60***	4.43
	*Qbafl.acs–2B.1*	Xbarc1072–Xwmc272	0	E2	–1.46***	3.05	–1.47***	3.26
	*Qbafl.acs–3A.1*	Xwmc695–Xgwm162	0	E1	–1.78***	3.43	–1.78***	6.91
				E3	–1.35***	3.71		
				E4	–1.54***	2.86	–1.58***	4.57
	*Qbafl.acs–4D.1*	Xwmc473–Xwmc489	0	E2	–1.75***	2.18	–1.64***	3.83
	*Qbafl.acs–5B.1*	Xbarc4–Xwmc376	0	E3	–1.28***	2.36		
	*Qbafl.acs–5B.2*	Xgwm499–Xwmc734	2	E3	–1.51***	3.30	–1.63***	3.14
	*Qbafl.acs–5B.3*	Xbarc164–Xbarc4	2	E1	–1.94***	4.01	–2.02***	3.95
				E2	–1.55***	2.89	–1.61***	2.75
	*Qbafl.acs–5B.4*	Xgwm408–Xwmc75	2	E4	–1.37***	2.45	–1.29***	2.86

*FLL* flag leaf length, *FLW* flag leaf width, *FLWR* flag leaf length to width ratio, *FLA* flag leaf area, *BAFL* basal angle of flag leaf. E1 to E4 represent the location at Yongdeng (103°18’ E, 36°42’ N), Gansu, China, in 2011and 2012, at Anning (103°51’ E, 36°04’ N), Gansu, China, in 2012, and at Yuzhong (104°07’ E, 35°51’ N), Gansu, China, in 2013, respectively. Position (cm): genetic distance from the left flanking marker in the marker interval. *A*: the additive effect; a positive value indicates the Q9086 allele having an increasing effect on the trait and a negative value represents the Longjian 19 allele having a decreasing effect. *h* ^2^(*A*) (%): the proportion of phenotypic variance explained by additive QTL. *AE*: the additive QTL × environment interaction effect; a positive value indicates *AE* effect having an increasing effect on the trait value in WW conditions and a negative value means *AE* effect having a decreasing effect on the trait value in DS conditions. *h* ^2^(*AE*) (%) : the phenotypic variance explained by the *AE* effect. **P ≤0.005, *** *P* ≤0.001

The majority of A-QTLs (35 of 55, or 63.6 %) for FLM-related traits were identified in one environment. Among them, 11 loci (7 for FLWR) showed no water environmental interactions. This suggested that the A-QTLs for FLWR expressed only in one environment were more insensitive to water treatments than those for other traits. However, the other 24 loci also showed significant A-QEIs with water environments. Of these, 15 A-QEIs were associated with DS and their *AE* effects decreased phenotypic values, whereas 9 A-QEIs were involved with WW and their *AE* effects increased phenotypic values. The A-QEIs in both groups individually explained from 1.37 to 10.19 % and from 2.41 to 6.97 % of the phenotypic variation, respectively. This indicated that the capacity of DS to influence phenotypic variation in the traits was stronger than those of WW. In particular, *Qfll.acs-5A.1* made a greater contribution to phenotypic variation in FLL not only by *A* effect (9.78 %) but also by *AE* effect (10.19 %), whereas the *A* and *AE* actions of other loci for corresponding traits were considerably lower.

Twenty of 55 (36.4 %) A-QTLs were detected in more than two environments, suggestive of stability. All of these loci were involved in A-QEIs with water environments to different extents, individually accounting for phenotypic variation of 1.83 to 7.90 % by *A* effects and 2.43 to 9.83 % by *AE* effects. Each of these loci even showed the same direction of *A* or *AE* effects in responding to different environments. Two loci, *Qflwr.acs-3A.1* and *Qflw.acs-5A.1*, were repeatedly detected in all four environments, while both *A* effects contributed by Longjian 19 and *AE* effects associated with DS deceasing phenotypic values. Similarly, seven A-QTLs were identified in three environments, where all *AE* effects related to DS and deceased phenotypic values. However, the genetic sources of *A* effects differed from these loci. Five of these loci, *Qfll.acs-1B.1*, *Qflw.acs-5A.3*, *Qflwr.acs-5A*, *Qfla.acs-1B* and *Qfla.acs-5A*, had favorable alleles contributed by Q9086, whereas the other two, *Qfll.acs-4D.1* and *Qbafl.acs-3A.1*, had favorable alleles from Longjian 19. The remaining 11 stable A-QTLs were identified in two environments, and involved three combinations of *A* and *AE* effects. Five loci, *Qfll.acs-1B.2*, *Qfll.acs-2B.1*, *Qflw.acs-3A.1*, *Qflw.acs-5A.2* and *Qfla.acs-2B.1*, inherited their *A* effects from Q9086 and *AE* effects related to DS, which result was opposite to those of the other three loci, *Qfll.acs-5A.2*, *Qflw.acs-2A.1* and *Qflwr.acs-2B*. The remaining three loci, *Qflw.acs-2B.1*, *Qfla.acs-3A* and *Qbafl.acs-5B*.3, possessed *A* effects form Longjian 19 and *AE* effects involving DS.

### Epistatic QTLs and water environmental interactions

All traits related to FLM were significantly affected by *AA* and *AAE* effects. Fifty one pairs of AA-QTLs were identified on all chromosomes except 6D, accounting for phenotypic variations of 2.01 to 8.25 % in different traits and environments (Table 6). The numbers of epistatic pairs for each trait differed from 7 (FLA) to 15 (FLL). Only six significant A-QTLs participated in epistatic interactions. However, most epistatic interactions (94.1 %) involving individual components lacked significant *A* effects. Among them, 28 pairs had significant *AA* effects to increase phenotypic values, indicating that the parent-type effect was higher than the recombinant-type effect, whereas the other 23 pairs showed *AA* effects decreasing phenotypic values where recombinant-type effects were higher than parent-type effects. By contrast to other traits, FLW showed remarkable disequilibrium between the two types of *AA* effects, because most of the epistatic pairs (75 %) enhanced *AA* effects to increase phenotypic values.

**Table 6:** Epistatic effects and interacting effects of epistatic QTL × environment of identified QTL for traits related to flag leaf morphology of main shoots in the RIL population

Trait	QTL_*i*_	Flanking marker	Position(cM)	QTL_*j*_	Flanking marker	Position (cM)	Environ.	*AA*	*h* ^2^(*AA*)%	*AAE*	*h* ^2^(*AAE*)%
FLL	*Qfll.acs–1A.1*	Xmag1022–Xwmc24	0	*Qfll.acs–1A.2*	Xwmc93–Xcfd25	0	E3	0.55***	8.25	–0.25**	3.50
	*Qfll.acs–1B.1*	Xwmc694–Xwmc156	0	*Qfll.acs–2D.1*	Xmag1280–Xgwm157	2	E1	–0.37***	2.43		
	*Qfll.acs–1B.3*	Xwmc830–Xwmc44	2	*Qfll.acs–3B.2*	Xgwm566–Xwmc231	2	E3	–0.30***	2.51		
	*Qfll.acs–1D*	Xbarc169–Xwmc216	2	*Qfll.acs–7A*	Xwmc596–Xgwm260	2	E4	0.34***	2.06	–0.39***	2.94
	*Qfll.acs–2A.1*	Xwmc474–Xwmc296	4	*Qfll.acs–4D.4*	Xwmc399–Xwmc622	2	E3	0.35***	3.43		
	*Qfll.acs–2A.2*	Xgwm122–Xmag2150	0	*Qfll.acs–4A.3*	Xwmc757–Xgwm613	0	E4	–0.42***	3.27	0.33***	4.05
	*Qfll.acs–2B.2*	Xbarc167–Xmag3698	0	*Qfll.acs–6B.2*	Xbarc24–Xpsp3131	4	E3	0.41***	4.67	–0.32***	4.08
	*Qfll.acs–2D.1*	Xmag1280–Xgwm157	2	*Qfll.acs–3A.4*	Xwmc695–Xgwm162	0	E1	–0.40***	2.93	–0.36***	2.42
	*Qfll.acs–3A.3*	Xwmc11–Xgwm391	6	*Qfll.acs–5A.4*	Xgwm186–Xcfa2185	0	E4	–0.49***	4.35	–0.50***	5.11
	*Qfll.acs–3B.1*	Xwmc236–Xmag3356	0	*Qfll.acs–4A.4*	Xgwm397–Xgwm613	0	E2	–0.36***	2.78		
	*Qfll.acs–4A.2*	Xgwm160–Xwmc497	4	*Qfll.acs–7B*	Xwmc526–Xwmc232	2	E1	–0.47***	3.95	–0.41***	3.86
	*Qfll.acs–4B*	Xbarc1133–Xbarc90	4	*Qfll.acs–1A.3*	Xgwm135–Xwmc304	0	E4	–0.45***	3.73	0.31***	3.42
	*Qfll.acs–4D.2*	Xwmc489–Xgdm61	0	*Qfll.acs–1A.4*	Xwmc20–Xbarc240	2	E1	0.36***	3.61	–0.29***	4.73
							E3	0.42***	4.71		
	*Qfll.acs–4D.3*	Xwmc473–Xwmc489	0	*Qfll.acs–2A.3*	Xgwm558–Xbarc208	8	E4	0.35***	2.21		
	*Qfll.acs–6A.1*	Xwmc553–Xwmc179	0	*Qfll.acs–6A.2*	Xbarc113–Xwmc621	0	E4	0.40***	2.91		
FLW	*Qflw.acs–1A.1*	Xgwm33–Xwmc818	0	*Qflw.acs–1A.2*	Xwmc611–Xwmc20	12	E3	0.03***	2.01	0.02***	3.41
	*Qflw.acs–1B*	Xbarc131–Xgwm413	2	*Qflw.acs–3B.1*	Xgwm108–Xpsp3035	0	E3	–0.03***	2.12	–0.03***	2.35
	*Qflw.acs–1D*	Xwmc429–Xwmc339	4	*Qflw.acs–7A.2*	Xksum153–Xwmc607	6	E2	–0.02***	2.36		
	*Qflw.acs–2A.3*	Xgwm512–Xgwm30	0	*Qflw.acs–5B.3*	Xmag959–Xwmc740	0	E4	0.02***	2.05	–0.02***	2.28
	*Qflw.acs–3A.3*	Xwmc505–Xwmc343	0	*Qflw.acs–2D*	Xwmc112–Xgwm484	0	E4	0.02***	2.27		
	*Qflw.acs–3A.4*	Xwmc50–Xksum222	6	*Qflw.acs–3B.2*	Xwmc366–Xgdm64	0	E2	0.03***	3.05		
	*Qflw.acs–4A*	Xgwm613–Xmag3733	10	*Qflw.acs–7D*	Xgdm67–Xmag892	0	E2	0.02***	2.36	–0.02***	2.50
	*Qflw.acs–5A.1*	Xwmc492–Xgwm96	0	*Qflw.acs–5B.3*	Xmag959–Xwmc740	2	E1	0.02***	2.05	0.01***	2.23
FLWR	*Qflwr.acs–1A.1*	Xcfa2513–Xksum104	0	*Qflwr.acs–4D*	Xwmc399–Xwmc622	0	E1	0.20***	2.18	0.16**	2.84
	*Qflwr.acs–1A.2*	Xgdm36–Xbarc83	0	*Qflwr.acs–7D*	Xgwm121–Xgdm67	0	E2	0.29***	4.18	–0.31***	4.62
	*Qflwr.acs–1B.2*	Xbarc61–Xwmc134	10	*Qflwr.acs–2D.2*	Xmag1280–Xgwm157	0	E1	–0.29***	4.56		
	*Qflwr.acs–2B*	Xwmc272–Xgwm630	0	*Qflwr.acs–4B.2*	Xbarc90–Xgwm540	2	E3	–0.23***	2.86	–0.19***	2.82
	*Qflwr.acs–2D.1*	Xwmc243–Xcfd239	0	*Qflwr.acs–5D*	Xcfd183–Xwmc212	2	E2	–0.38***	7.28	–0.30***	4.99
	*Qflwr.acs–3A.3*	Xwmc264–Xgwm494	0	*Qflwr.acs–4A.2*	Xgwm613–Xmag3733	6	E1	0.39***	7.67		
							E2	0.29***	4.36		
	*Qflwr.acs–3B.1*	Xbarc173–Xgwm284	0	*Qflwr.acs–1B.3*	Xgwm413–Xwmc419	12	E3	0.39***	5.93	–0.40***	5.05
	*Qflwr.acs–3B.2*	Xgdm64–Xwmc51	0	*Qflwr.acs–3A.4*	Xgwm67–Xwmc264	2	E4	0.21***	2.45	–0.23***	2.64
	*Qflwr.acs–4A.1*	Xwmc757–Xgwm610	2	*Qflwr.acs–4A.3*	Xgwm397–Xgwm613	0	E4	–0.26***	3.73	–0.22***	3.20
	*Qflwr.acs–4B.1*	Xksum244–Xmag2055	2	*Qflwr.acs–5B*	Xcfd10–Xbarc59	2	E4	–0.23***	2.73	0.18**	3.31
	*Qflwr.acs–6A.2*	Xgwm169–Xwmc580	2	*Qflwr.acs–7B*	Xbarc315–Xwmc311	2	E4	0.21***	2.29		
	*Qflwr.acs–6B.1*	Xwmc539–Xmag590	0	*Qflwr.acs–6B.2*	Xbarc79–Xgwm626	0	E2	0.31***	4.95	–0.35***	4.27
FLA	*Qfla.acs–1A.1*	Xwmc104–Xcfa2219	14	*Qfla.acs–1D*	Xcfd72–Xwmc429	0	E2	0.62***	3.08	–0.35***	1.93
	*Qfla.acs–2A.1*	Xwmc474–Xwmc296	2	*Qfla.acs–2B.2*	Xgwm55–Xbarc128	0	E1	–1.39***	2.61	0.66***	1.18
	*Qfla.acs–2A.2*	Xgwm249–Xcfa2263	0	*Qfla.acs–4D.3*	Xgdm61–Xwmc457	18	E3	–0.74***	2.33	–0.68***	2.51
	*Qfla.acs–2B.1*	Xbarc1072–Xwmc272	0	*Qfla.acs–6A.2*	Xbarc113–Xwmc621	0	E3	1.16***	5.75	–0.94***	6.02
	*Qfla.acs–3D*	Xwmc43–Xwmc675	16	*Qfla.acs–5D*	Xcfd183–Xwmc212	4	E3	0.77***	3.56	–0.73***	3.34
	*Qfla.acs–4A*	Xmag3733–Xwmc707	0	*Qfla.acs–7D*	Xgdm67–Xmag892	0	E2	0.57***	2.58	–0.62***	2.85
	*Qflaacs–5A*	Xwmc205–Xgwm154	4	*Qfla.acs–1A.2*	Xgwm135–Xwmc304	0	E4	–0.87***	3.27		
BAFL	*Qbafl.acs–1D*	Xbarc169–Xwmc216	4	*Qbafl.acs–7A.1*	Xwmc596–Xgwm260	0	E3	1.79***	2.03	–1.85***	2.18
							E4	2.72***	4.72	–1.84***	4.31
	*Qbafl.acs–2B.2*	Xbarc1155–Xcfd73	4	*Qbafl.acs–3B.2*	Xbarc68–Xgwm285	0	E1	1.94***	2.61	–1.75***	2.48
	*Qbafl.acs–3A.1*	Xwmc695–Xgwm162	0	*Qbafl.acs–2B.3*	Xgwm132–Xcfa2278	0	E2	–2.64***	2.58		
	*Qbafl.acs–3B.1*	Xbarc1077–Xwmc366	2	*Qbafl.acs–7A.2*	Xgwm282–Xmag828	4	E2	–2.35***	2.05	–1.67***	2.06
	*Qbafl.acs–4A.1*	Xwmc420–Xgwm601	2	*Qbafl.acs–4A.2*	Xgwm610–Xgwm397	2	E4	–1.85***	2.19	–1.72***	2.34
	*Qbafl.acs–4B.1*	Xksum238–Xcfd39	0	*Qbafl.acs–4D.2*	Xksum180–Xwmc48	10	E1	–1.93***	2.57		
	*Qbafl.acs–4B.2*	Xbarc292–Xbarc1133	0	*Qbafl.acs–1A*	Xbarc197–Xcfa2513	0	E4	–2.38***	3.62	–1.83***	4.29
	*Qbafl.acs–5A*	Xwmc630–Xmag4263	2	*Qbafl.acs–3B.3*	Xwmc291–Xgwm108	6	E3	2.37***	5.72	–1.94***	4.32
	*Qbafl.acs–6A*	Xgwm427–Xwmc642	0	*Qbafl.acs–3A.2*	Xwmc532–Xgwm674	14	E1	1.83***	2.32		

*FLL* flag leaf length, *FLW* flag leaf width, *FLWR* flag leaf length to width ratio, *FLA* flag leaf area, *BAFL* basal angle of flag leaf. E1 to E4 represent the location at Yongdeng (103°18’ E, 36°42’ N), Gansu, China, in 2011and 2012, at Anning (103°51’ E, 36°04’ N), Gansu, China, in 2012, and at Yuzhong (104°07’ E, 35°51’ N), Gansu, China, in 2013, respectively. QTL_*i*_ and QTL_*j*_ are a pair of QTL detected by two–dimensional searching. *AA*: the direction of the epistatic effect; a positive value means that the parent–type effect is greater than the recombinant–type effect and a negative value means that the parent–type effect is less than the recombinant–type effect. *h* ^2^(*AA*) (%) : the phenotypic variance explained by epistatic QTL. *AAE*, a positive value indicates *AAE* effect having an increasing effect on the trait value in WW conditions and a negative value means *AAE* effect having a decreasing effect on the trait value in DS conditions. *h* ^2^(*AAE*) (%):the phenotypic variance explained by *AAE* effect. **P ≤0.005, ***P ≤0.001

Of the putative AA-QTLs, 48 pairs for five traits were identified in single environment, whereas only three pairs, including each pair for FLL, FLWR and BAFL, were repeatedly detected in two environments. This suggested that expressions of epistatic loci for FLM-related traits were more sensitive to environments than those of additive loci. Furthermore, 33 pairs with significant *AA* effects involved significant E-QEIs under two water regimes in single environment. Regarding each trait, 53.3 % (FLL) to 85.7 % (FLA) of the AA-QTLs significantly participated in E-QEIs. Among them, 26 E-QEIs with *AAE* effects decreasing phenotypic values were associated with DS, individually explaining 1.93 to 6.02 % of the phenotypic variation. The other seven E-QEIs with *AAE* effects increasing phenotypic values were involved in WW, individually accounting for phenotypic variation of 1.18 to 4.05 %. With the exception of one stable epistatic pair (*Qflwr.acs-3A.3* × *Qflwr.acs-4A.2*) for FLWR without any E-QEIs, the other two stable epistatic pairs reacted to DS and thus exhibited *AAE* effects to decrease the phenotypic values, individually explaining 2.18 to 4.73 % of the phenotypic variation. This result also showed that the DS had a stronger impact than the WW on *AAE* effects, consistent with the case of *AE* effects.

### Chromosomal distribution and genetic contributions of detectable QTLs

**Fig. 1:**
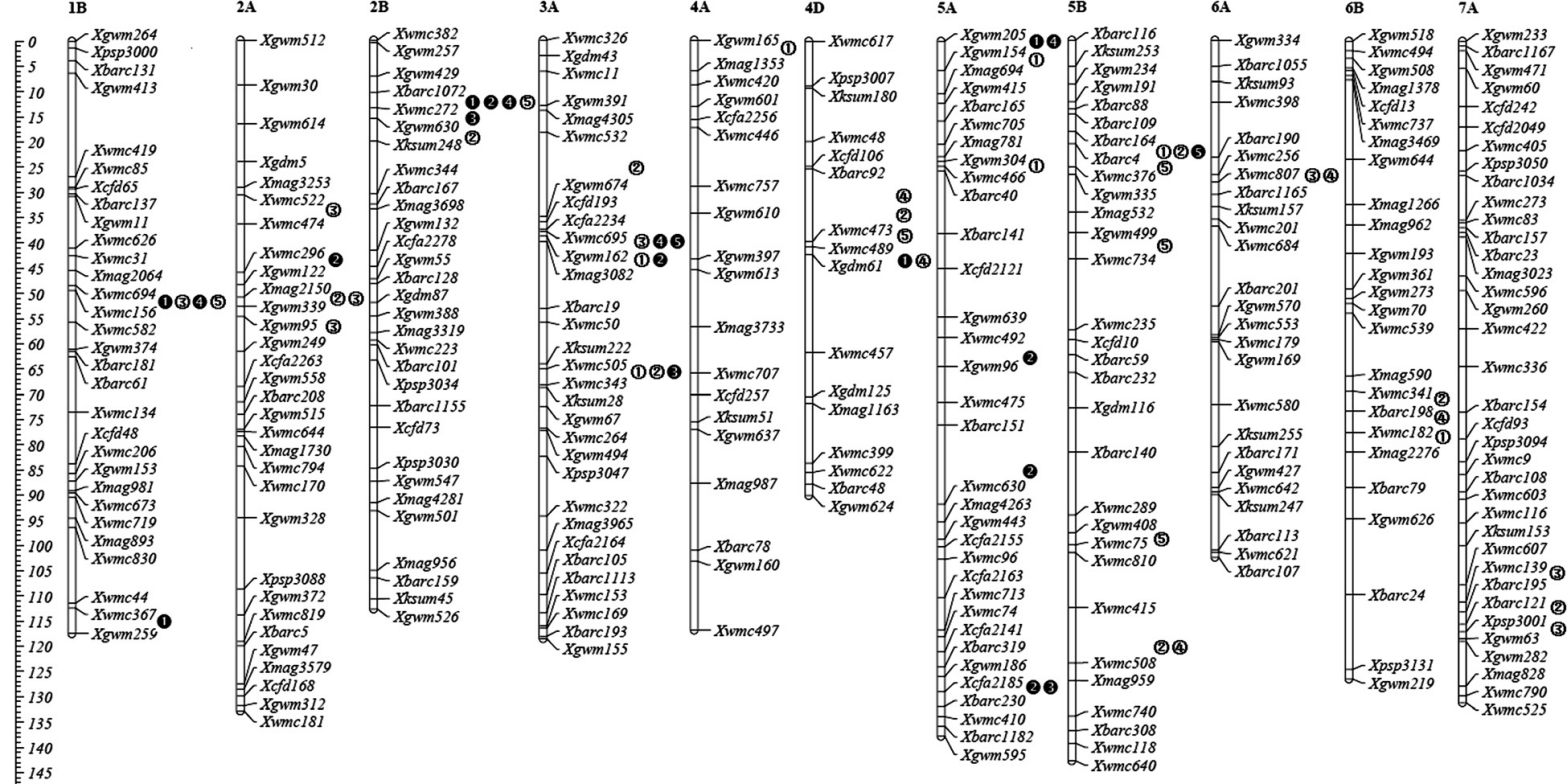
Chromosome locations of A-QTLs for traits related to flag leaf morphology of main shoots in wheat RIL population. **①**, **②**, **③**, **④** and **⑤** showed A-QTLs identified in a specific environment for flag leaf length (FLL), flag leaf width (FLW), flag leaf length to width ratio (FLWR), flag leaf area (FLA) and basal angle of flag leaf (BAFL), respectively. ➊, ➋, ➌, ➍ and ➎ represented A-QTLs identified in two or more environments for FLL, FLW, FLWR, FLA and BAFL, respectively

In this study, 55 significant A-QTLs for FLM-related traits in the RIL population were mapped on 11 chromosomes. They were more frequently located on chromosomes 1B, 2A, 2B, 3A, 4D, 5A and 5B (more than 5 A-QTLs). The highest number (9 or 16.4 %) was detected on chromosome 3A, whereas the lowest number (1 or 1.8 %) was on chromosome 4A. Chromosomes 2B and 3A possessed A-QTLs for all tested traits. An interesting feature was the highly concentrated distribution of A-QTLs in a few chromosomal regions and the existence of QTL hotspots, namely, the chromosomal regions shared by multiple QTL (Table 5, Fig. 1). For example, several A-QTLs underlying FLL, FLWR, FLA and BAFL were detected within the marker interval Xwmc694- Xwmc156 on chromosome 1B. Similarly, A-QTLs for FLL, FLW, FLA and BAFL were co-located in the marker interval of Xbarc1072-Xwmc272 on chromosome 2B. The other ten specific intervals, for example, Xmag2150-Xgwm339 on 2A, Xwmc695-Xgwm162, Xgwm162-Xmag3082 and Xwmc505-Xwmc343 on 3A, and so on, harbored A-QTLs controlling two to three traits. On the other hand, QTL clustering also occurred in several neighboring marker intervals. For example, the region flanking markers from Xwmc522 to Xgwm249 on chromosome 2A was shared by A-QTLs associated with FLW and FLWR. A-QTLs for all five traits shared neighboring intervals Xbarc1072 to Xksum248 on chromosome 2B and Xwmc695 to Xmag3082 on chromosome 3A. The other clustered A-QTLs involving two to four traits were mapped in five adjacent marker intervals Xbarc92 to Xgdm61 on chromosome 4D, Xgwm205 to Xmag694 on chromosome 5A, Xbarc164 to Xwmc376 on chromosome 5B, Xwmc341 to Xmag2276 on chromosome 6B, and Xwmc139 to Xgwm63 on chromosome 7A. This indicated that specific hotspot regions might carry genes controlling traits contributing to FLM.

**Fig. 2:**
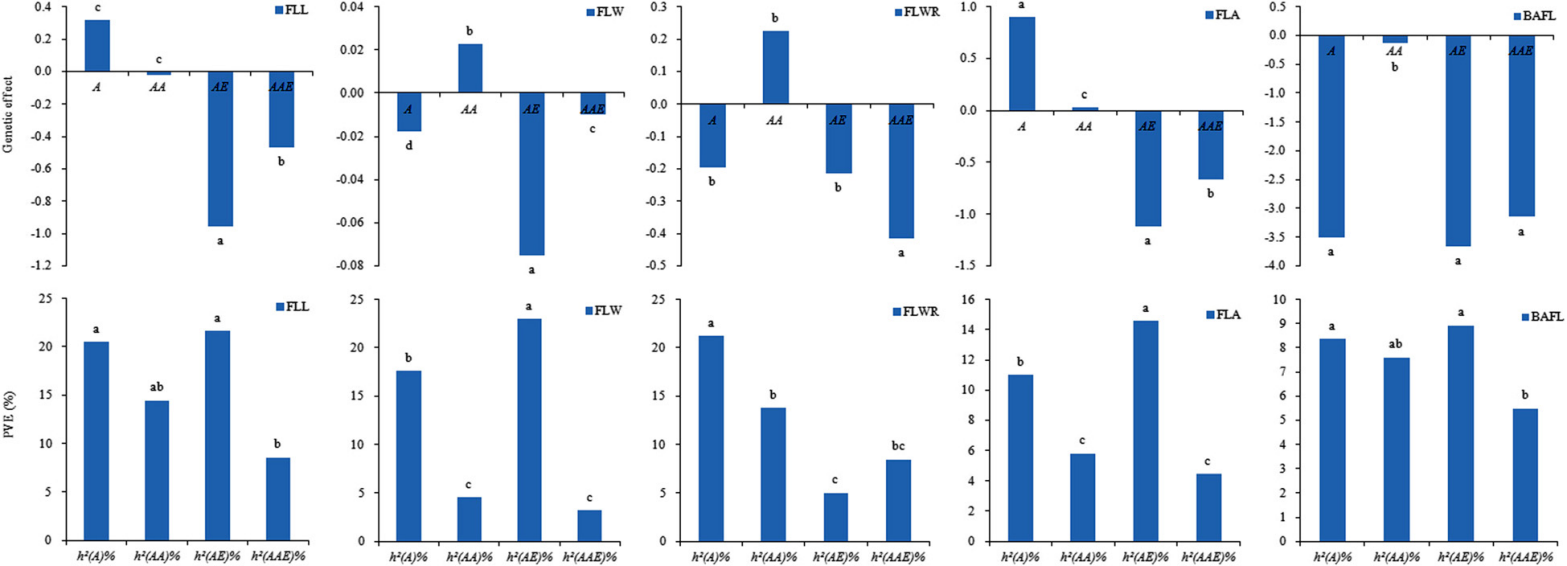
Genetic effects and phenotypic variation explained (PVE) by genetic components for traits related to flag leaf morphology of main shoots in wheat RIL population across four environments. *A*, *AA*, *AE* and *AAE* mean additive effect, epistatic effect, additive QTL × environment interaction effect and epistatic QTL × environment interaction effect, respectively; *h* ^2^(*A*), *h* ^2^(*AA*), *h* ^2^(*AE*) and *h* ^2^(*AAE*) represent phenotypic variation explained (PVE) by *A*, *AA*, *AE* and *AAE*, respectively. Different lowercase letters indicate significant differences (*P* < 0.05) between genetic components for each trait

The mean genetic component effects and phenotypic variations explaining genetic effects for all tested traits across environments E1 to E4 are given in Fig. 2. Both above mean values significantly differed from genetic components for each trait. Genetic effects generally acted to decrease phenotypic values. In this case, the highest values of genetic effects were highlighted in *AE* and/or *AAE*, although the *A* effects for FLWR, FLA and BAFL were also important. Thus based on effect magnitudes of genetic component effects, it could be perceived that genetic regulation of FLM was more ascribable to QEIs effects caused by DS, rather than additive and epistatic effects. In addition, the means of phenotypic variations explained by genetic effects also further illustrated the characteristics of QTL expressions for tested traits. By contrast, the contribution rates of phenotypic variation explained by both *A* and *AE* effects for FLL and BAFL predominated over those explained by *AA* and *AAE* effects. Similarly, the predominant effects were contributed by *AE* effect for FLW and FLA and by *A* effect for FLWR. It was obvious that the magnitudes of genetic effects were inconsistent with those of their corresponding genetic contribution rates. On the whole, although the importance of genetic components differed from tested traits, additive and QEIs effects predominated in governing the phenotypic variation in FLM.

## Discussion

### Phenotypic variations in response to drought stress

The flag leaf is the most important source organ for synthesis and output of assimilates during the reproductive stage, and is responsible for regulating final plant growth and yield formation in cereal crops [4, 5]. The morphological attributes of flag leaves, such as FLL, FLW, FLA and BAFL, are therefore critical factors in determining a desirable plant type [43], and also sense environmental signals for adaptation [4, 5]. In this study, ANOVA clearly showed that phenotypic means of tested traits in a RIL population were more affected by both water regime and environment factors. The phenotypic means under the DS were significantly lower than those under the WW conditions (Tables 1 and 2). These indicated that flag leaves remained smaller sizes and erect postures when adapting to DS, in agreement with previous studies [19–21]. Obviously, reduced flag leaf size should be beneficial in limiting excessive water losses by transpiration [17], while maintaining assimilate synthesis and transport to grain as efficiently as possible [18].

Most traits related to FLM were positively correlated with each other in both water regimes, whereas correlation coefficients under DS (*r* = 0.31^*^to 0.93^**^) were generally higher those under WW conditions (*r* = 0.29^*^to 0.81^**^) (Table 3). This suggested that all components related to FLM under DS might be more effectively coordinated by phenotypic reduction to withstand adverse conditions. By contrast, FLL appeared to be the main contributor to FLA and also influenced BAFL to some extent, as evidenced by higher correlation with each other. However, when working with a wheat RIL population (Kenong 9204 × Jing 411) under nitrogen stress, Fan et al. [32] found that the positive correlation between FLW and FLA (0.84^**^) was stronger than that between FLL and FLA (0.57^**^), suggesting a predominant contribution of FLW relative to FLA [32]. This indicated that water and nitrogen supply could affect flag leaf size and shape in different ways. Of course, this possibility cannot be excluded from the differences in the genetic backgrounds of the two populations. FLL and FLA showed higher and more significant positive correlations with PH, KW and YP than with other traits under both water regimes across environments (Table 4), indicating that FLL and FLA contributed more to PH, KW and YP.

### Genetic components and QTL-by-environment interactions

Although a wealth of information from previous studies considerably improved our understanding of the morpho-physiological functions of flag leaves [4, 5], as well as applications in wheat breeding programs [4, 19], few studies considered the genetic basis of FLM-related traits under water-deficit conditions at the molecular level [15, 24, 29, 31, 32]. The present study evaluated the genetic basis of FLL, FLW, FLWR, FLA and BAFL in a wheat RIL population of 120 lines under two water regimes over four environments that differed in the amounts of available water. An important aspect of the study was the use of composite interval mapping of a mixed linear model to permit division of genetic effects into genetic main effects (*A* and *AA*) and QEIs (*AE* and *AAE*) effects. So far, few studies on the QTL identification for FLM separated genetic interactions from epistasis and QEIs [15, 24, 28–32]. When QTL analyses ignore genetic component interactions, it leads to biased estimates of main-effect QTLs and affects the accuracy of isolating main-effect QTLs [44]. In this regard, the importance of epistasis and QEIs in determining the quantitative genetic basis of other traits in wheat, such as yield-related and physiological traits, has been documented [45, 46]. These studies showed that the actions of QTLs with additive effects were not completely independent, but varied depending upon their interactions with other loci and/or with environmental factors. Our study also confirmed that phenotypic variation of all traits was controlled by *A* and *AA* effects, as well as QEIs (*AE* and *AAE*) effects (Tables 5 and 6). As genetic main effects, *A* and *AA* effects were largely responsible for the genetic basis of FLM, but the cumulative contributions from *AA* effects were significantly lower than those from *A* effects for all tested traits (Fig. 2). The results were consistent with the previous findings involved in yield-associated traits in other cereal crops [47, 48]. It was interpreted that low contributions to phenotypic variance explained by *AA* effects were due to large numbers of AA-QTLs with minor genetic effects [46]. On the other hand, we concluded that the phenotypic variation in FLM was predominantly controlled by additive and QEIs effects, depending on exclusive genetic contributions.

Genotype × environment interaction is critical in determining the adaptation and fitness of genotypes in adverse environments [47], resulting in phenotypic variation referred to as phenotypic plasticity [49]. The phenotypic plasticity of quantitative traits arises in nature from interactions between QTLs and environments at the molecular level [50]. Numerous cases of such QEIs for agronomic and physiological traits showed that QTL expressions varied across environments [45, 46, 49, 50]. In the present study, A-QEIs and E-QEIs for all five traits were also identified. For example, 80 % (44 of 55) of A-QTLs and 68.6 % (35 of 51) of AA-QTLs participated in QEIs, of which 72.7 % A-QEIs and 80 % E-QEIs were associated with DS, individually explaining 1.37 to 10.19 % and 1.93 to 6.02 % of the phenotypic variation, respectively (Tables 5 and 6). This indicated that DS influenced the phenotypic variation in these traits more strongly than WW conditions. Moreover, these QEIs effects under DS decreased phenotypic values of FLM. This also seemed to explain why FLM-related traits showed higher coefficients of variation (13.51 to 38.25 %) and lower phenotypic values under DS, compared to those under WW conditions (Table 1). The present study also suggested that QTLs for FLM-related traits could have different expression patterns responsive to different environments, because a majority of them were detected in single environment. Similar results were obtained for other quantitative traits such as grain yield and related traits in rice [51] and wheat [45, 46]. Li et al. [51] suggested that this phenomenon might occur in any of the following situations: (1) a QTL expressed in one environment but not in another, as reflected by inconsistent detection of QTL across environments; (2) a QTL expressed strongly in one environment but weakly in another, as indicated by variation in its effects across environments; and (3) a QTL expressed very differently and with opposite effects in different environments [51].

### Chromosomal location and pleiotropy of QTLs

In accord with previous studies [15, 24, 29, 30, 32], the distributions of A-QTLs controlling FLM-related traits in the present work behaved in a highly uneven way (Fig. 1). They were more frequently located on chromosomes 1B, 2A, 2B, 3A, 4D, 5A and 5B (more than 5 A-QTLs for each chromosome). The highest number of QTLs (9 or 16.4 %) was detected on chromosome 3A, whereas the lowest (1 or 1.8 %) was on chromosome 4A. Chromosomes 2B and 3A possessed A-QTLs for all tested traits. Similar results were also observed by Wu et al. [24]. This indicated that these important chromosomes carried large numbers of genes controlling FLM. Furthermore, QTLs for FLM-related traits were likewise highly concentrated in a few chromosomal regions on the same chromosomes (Fig. 1). These QTL clusters were generally involved in correlated traits with higher correlation coefficients between traits (Table 3), similar to the previous studies [11, 12, 24, 33]. It was hypothesized that the inheritance of component traits of FLM could be highly correlated with each other, and even with yield-related traits, because many specific or adjacent intervals with QTLs for traits associated with FLM share locations with QTLs for yield-related traits in wheat [28, 29, 31, 32] and rice [5, 11, 12]. Using the same RIL population in our previous studies, some reported QTLs for PH [38] and thousand-grain weight (TGW) [39] were co-located or adjacent the locations of the present QTLs for FLM-related traits in particular marker intervals on chromosomes 2A, 2B, 5B and 7A. Moreover, some reported QTLs for heading date were shared the same marker interval Xbarc151-Xwmc630 on chromosome 5A with stable QTL for FLW, Xbarc109-Xwmc376 on chromosome 5B with QTLs for FLL, FLW and BAFL [52], and Xgwm408-Xwmc75 on chromosome 5B with QTL for BAFL [52, 53]. However, it remains a puzzling question whether these clustered QTLs represent close linkages of multiple genes affecting different traits or have pleiotropic effects of regulatory genes that affect the related traits [12]. One particular interpretation is that the nature of QTL clusters in particular chromosomal regions might be resolved by increasing population size, or by using overlapping substitution lines. As a result, most of QTL clusters for correlated quantitative traits were proved to inherit as a linkage way, instead of pleiotropy [12].

### Stable QTLs compared with previous findings

Twenty of 55 A-QTLs (36.4 %) for five traits related to FLM were repeatedly detected in more than two environments, suggestive of stable A-QTLs (Table 5). Of these, *Qflwr.acs-3A.1* and *Qflw.acs-5A.1* were continuously active across all four environments, whereas the other 18 loci were found in two to three environments. These stable QTLs provide useful information for genetic improvement of flag leaf morphological traits in wheat through QTL pyramiding. By using a wheat microsatellite consensus map [54] as a reference map, some QTLs controlling FLM-related traits in the present work were mapped to the same or similar chromosomal regions to previous studies. For example, the location of a stable A-QTL for FLL, *Qfll.acs-1B.2*, in the marker interval Xwmc367-Xgwm259 on chromosome 1B, overlapped the location of a QTL for FLL reported by Ma et al. [37]. Marker interval of Xwmc694-Xwmc156 on chromosome 1A co-located stable QTLs for FLL, FLA, FLWR and BAFL was earlier reported as a locus for FLL [28]. *Qflwr.acs-2A.2* for FLWR in marker interval Xwmc522-Xwmc474 on chromosome 2A overlapped a BAFL QTL reported by Isidro et al. [15]. Several previous studies reported QTL for FLW on chromosome 5A [24, 29, 30, 37] in similar position in the present study. A stable QTL for FLW, *Qflw.acs-5A.1*, in marker interval Xwmc492-Xgwm96 in chromosome 5A was near to a fine-mapped locus for FLW reported by Xue et al. [30]. The other two stable QTLs, *Qflw.acs-5A.2* for FLW and *Qflwr.acs-5A* for FLWR in marker interval Xcfa2185-Xbarc230, were possibly the same as QTLs for FLW reported by Ma et al. [37] and Jia et al. [29], because of proximity to Xcfa2185. *Qflw.acs-5A.3* for FLW was mapped to a similar position to another reported locus for FLW in marker interval of Xbarc151-Xwmc630 [29]. The remaining loci on chromosome 5A, *Qfll.acs-5A.1*, *Qfll.acs-5A.2* and *Qfll.acs-5A.3* for FLL, and *Qfla.acs-5A* for FLA, were co-located or adjacent to the corresponding loci governing FLW and FLA identified by Wu et al. [24]. Likewise, we mapped eight QTLs for all FLM-related traits, except FLWR, in four marker intervals in chromosome 5B, of which Xgwm499-Xwmc734 and Xgwm408-Xwmc75 overlapped or were adjacent to the locations of QTLs for BAFL detected earlier [15]. These common QTLs and linked molecular markers should be useful for MAS designed to improve flag leaf size and shape, along with yield potential under drought conditions. Furthermore, the development of near-isogenic lines and enlargement of population sizes for genetic analysis should help to resolve whether QTL clusters represent linkage of independent genes or pleiotropy [12].

## Conclusions

We found that flag leaf morphology in wheat was mainly controlled by additive and QEIs effects, where more QEIs effects occurred in drought stress and depressed phenotypic performances. Several QTL cluster regions were suggestive of tight linkage or pleiotropy in the inheritance of tested traits. Twenty stable QTLs for flag leaf morphological traits could be useful for the genetic improvement of drought tolerance in wheat through QTL pyramiding.

## Abbreviations

*A*, additive effect; *AA*, epistatic effect; *AAE*, epistatic QTL × environment interaction effect; AA-QTLs, epistatic QTLs; *AE*, additive QTL × environment interaction effect; ANOVA, analysis of variance; A-QEIs, additive QEIs; A-QTLs, additive QTLs; BAFL, basal angle of flag leaf; CV, coefficients of variation; DH, double haploid; DS, drought stressed; E-QEIs, epistatic QEIs; FLA, flag area; FLL, flag length; FLM flag leaf morphology; FLW, flag width; FLWR, flag leaf length to width ratio; *h*^2^_B_, broadsense heritability; KN, kernel number; KW, kernel weight per spike; LOD, log odds; PH, plant height; QEIs, QTL × environment interactions; QTLs, quantitative trait loci; *r*, correlation coefficients; RIL, recombinant inbred line; SN, spikelet number; SSR, simple sequence repeats; TGW, thousand-grain weight; WW, well-watered; YP, yield per plant.
